# ACKNOWLEDGEMENT TO REVIEWERS

**DOI:** 10.2174/138920292605250905145827

**Published:** 2025-09-05

Bentham Science Publishers would like to thank and appreciate the co-operation from all reviewers for their constructive comments and feedback on the manuscripts submitted to **Current Genomics**. Their efforts have contributed greatly to the high quality and continuous growth of the journal. Given below is the list of reviewers who reviewed articles for the Journal during 2025:

Sheraz Ahmad (China)

Waleed H. Almalki (Saudi Arabia)

Yan An (China)

Ahila Ashraf (India)

Yener Aydin (Turkiye)

Rostyslav V. Bubnov (Ukraine)

Zafir Buraei (USA)

Rafael Camacho-Carranza (Mexico)

Xiaoguang Chen (China)

Jinyan Chen (China)

Shan Chen (Singapore)

Minjie Chu (China)

Philip D. Cotter (USA)

Mehdi Dadashpour (Iran)

Shixue Dai (China)

Reza A. Darban (Iran)

Claudio De Felice (Italy)

Haixiao Dong (China)

Masoumeh Falah (Iran)

Denis S. Fedorinov (Russia)

Jose L. Fernandez-Garcia (Spain)

Ioannis A. Georgiou (Greece)

Rajarshi P. Ghosh (USA)

Bing Guan (China)

Qianjin Guo (China)

Minjie Hu (USA)

Chakresh K. Jain (India)

Cunmei Ji (China)

Shulei Jia (China)

Tingyun Jiang (China)

Yan-Jun Kang (China)

Saeid Kaviani (Iran)

Ajmal Khan (Pakistan)

Sulev V. Kõks (Australia)

Shubham Kumar (India)

Haixing Li (China)

Zhanchao Li (China)

Guocai Li (China)

Lizong Li (China)

Haobo Li (France)

Thomas Liehr (Germany)

Xiaoyan Liu (China)

Qian Liu (China)

Sergio Lopez-Briones (Mexico)

Yan Lu (China)

Fajin Lv (China)

Jinzhu Meng (China)

Akanksha Mishra (India)

Anuradha Muthukrishnan (India)

Pavel V. Nikitin (Russia)

Prince E. Norman (Sierra Leone)

Giovanni Pagano (Italy)

Jennifer Q. Pan (USA)

Manikantan Pappuswamy (India)

Elham Pashaei (USA)

Tippapha Pisithkul (Thailand)

Kaikai Qiao (China)

Bhuvnesh Rai (India)

Muhammad R. Rasyak (Indonesia)

Hyunju Ro (Korea, South)

Jagajjit Sahu (China)

Haojing Shao (China)

Akansha Singh (India)

Bowen Song (China)

Qianqian Song (USA)

Ricardo B. Souza (Brazil)

Tetsuro Taki (Japan)

Iman Tavassoly (USA)

David W. Ussery (USA)

Wenqiu Wang (China)

Yue Wang (China)

Guiyu Wang (China)

Cailin Wang (China)

Yiliang Wei (China)

Xiangsheng Xiao (China)

Yalda Yazdani (Iran)

Xianfu Yi (China)

Zheng Yuan (China)

Linghua Zhu (China)

Wentao Zhu (China)

